# 

**Published:** 1906-10

**Authors:** 


### BOOKS RECEIVED.

A Manual of Chemistry, General, Medical, and Pharmaceutical, including the Chemistry of the U. S. Pharmacopeia. By John Attfield, F.R.S., Professor of Practical Chemistry to the Pharmaceutical Society of Great Britain, 1862-96. Edited by Leonard Dobbin, Ph.D., Lecturer on Chemistry in the University of Edinburgh. Nineteenth Edition. 12 mo, pp. 776. Philadelphia and New York: Lea Brothers & Co. 1906. (Price, $2.50).

---

A Manual of Otology by Gorham Bacon, A.B., M.D., Professor of Otology in the College of Physicians and Surgeons, New York. With an Introductory Chapter by Clarence John Blake, M.D., Professor of Otology in Harvard University. Fourth Edition. 12 mo., pp. 485. With 134 Illustrations and 11 Plates. New York and Philadelphia: Lea Brothers & Co. 1906. (Price, $2.25).

---

Progressive Medicine, Vol. VIII, No. 3, September, 1906. A Quarterly Digest of Advances, Discoveries and Improvements in the Medical and Surgical Sciences. Edited by Hobart Amory Hare, M.D., Professor of Therapeutics and Materia Medica in the Jefferson Medical College of Philadelphia. Octavo, 298 pages, 13 illustrations. Lea Brothers & Co., Philadelphia and New York. (Per annum, in four cloth-bound volumes, $9.00; in paper binding, $6.00; carriage paid to any address).

---

The Practical Medicine Series of Year Books. Ten volumes. Issued under the general editorial charge of Gustavus P. Head, M.D., Professor of Laryngology and Rhinology in the Chicago Post-Graduate Medical School. Vol VI. General Medicine. Edited by Frank Billings, M.D., and J. H. Salisbury, M.D. Chicago: The Year Book Publishers. 1906. (Price: $1.25; entire series, $10.00).

---

American Practice of Surgery. A Complete System of the Science and Art of Surgery by Representative surgeons of the United States and Canada. Edited by Joseph D. Bryant, M.D., and Albert H. Buck, M.D., New York. Complete in eight volumes. Vol. I. Imperial octavo, pp. 818, with 294 illustrations in the text. New York: William Wood and Company. 1906.

---

A Textbook of Physiology. By Dr. Robert Tigerstedt, professor of physiology in the University of Helsingfors, Finland. Translated from the third German edition and edited by John R. Murlin,

A.M., Ph.D., Assistant Professor of Physiology in the University and Bellevue Hospital Medical College, New York. With an introduction in English by Professor Graham Lusk Ph.D., F.R.S. (Edin.). Octavo, pp. 751. New York: D. Appleton and Company. 1906. (Price: Cloth, $4.00).

---

Transactions of the Southern Surgical and Gynecological Association. Vol. XVIII. Eighteenth Session, held at Louisville, Ky., December 12-14, 1905. Octavo, pp. 456. Edited by William D. Haggard, M.D., Secretary. Philadelphia: William A. Dornan. 1906.

---

The Practical Medicine Series of Year Books. Ten Volumes. Issued under the general editorial charge of Gustavus P. Head, M.D., Professor of Laryngology and Rhinology in the Chicago Post-Graduate Medical School. Vol. V, series of 1906. Obstetrics. Edited by Joseph B. De Lee, A.M., M.D., with the collaboration of D. Roehler, M.D., and Herbert M. Stowe, M.D. Chicago: The Year Book Publishers. 1906. (Price: $1.25; entire series, $10.00).

---

The Practical Medicine Series of Year Books. Ten volumes. Issued under the general editorial charge of Gustavus P. Head, M.D., Professor of Laryngology and Rhinology in the Chicago Post-Graduate Medical School. Vol. IV, series of 1906. Gynecology. Edited by Emilius C. Dudly, A.M., M.D., and C. von Bachellé, M.S., M.D. Chicago: The Year Book Publishers. (Price: $1.50; entire series, $10.00)

